# The exoskeletons are here

**DOI:** 10.1186/1743-0003-6-17

**Published:** 2009-06-09

**Authors:** Daniel P Ferris

**Affiliations:** 1 School of Kinesiology, University of Michigan, Ann Arbor, MI, USA; 2 Department of Biomedical Engineering, University of Michigan, Ann Arbor, MI, USA; 3 Department of Physical Medicine and Rehabilitation, University of Michigan, Ann Arbor, MI, USA

## Abstract

It is a fantastic time for the field of robotic exoskeletons. Recent advances in actuators, sensors, materials, batteries, and computer processors have given new hope to creating the exoskeletons of yesteryear's science fiction. While the most common goal of an exoskeleton is to provide superhuman strength or endurance, scientists and engineers around the world are building exoskeletons with a wide range of diverse purposes. Exoskeletons can help patients with neurological disabilities improve their motor performance by providing task specific practice. Exoskeletons can help physiologists better understand how the human body works by providing a novel experimental perturbation. Exoskeletons can even help power mobile phones, music players, and other portable electronic devices by siphoning mechanical work performed during human locomotion. This special thematic series on robotic lower limb exoskeletons and orthoses includes eight papers presenting novel contributions to the field. The collective message of the papers is that robotic exoskeletons will contribute in many ways to the future benefit of humankind, and that future is not that distant.

## Introduction

In 2004, Rodney Brooks, then director of the Massachusetts Institute of Technology Computer Science and Artificial Intelligence Laboratory and general robot guru, proclaimed that "the robots are here" [1]. He made the case that robots had infiltrated our homes and everyday lives to the extent that it was no longer appropriate to say "the robots are coming". Brooks also went on to state that, in his opinion, robots in 2004 were where personal computers were in 1978. That is, they were both located just before the exponential expansion of their ubiquitous deployment throughout our civilization. Brooks predicted that in 15 years (i.e. 2019), robots would be everywhere, just as personal computers were everywhere in 1993 [1].

Adding a corollary onto Brooks' prediction, I firmly believe that robotic exoskeletons are today where computers were in 1978. Currently, popular media outlets routinely herald new robotic exoskeletons such as HAL, BLEEX, and XOS [2]. Even more recently, Honda has come out with variations, their Stride Management Assist and their Bodyweight Support Assist [3]. By 2024, people will be walking down the street, in the malls, and to their homes wearing robotic exoskeletons. They will make it easier for people to carry backpacks and walk for a long duration, and they will be portable, svelte, and fashionable. Rehabilitation clinics will have an assortment of exoskeletons available to aid patients that have experienced spinal cord injury, stroke, and other neurological disorders. There will be some models designed for assistive technology such that the patients will wear them anytime they walk, and there will be some models designed for rehabilitation such that they will be used for motor re-training in the clinic. There will also be various exoskeletons available that do not add mechanical power to the wearer, but harvest energy from the walking motion to power mobile phones and other portable electronic devices.

Exoskeleton development has an advantage over robot development in general because exoskeletons can rely on the intelligence of the human user. Exoskeletons can take advantage of all the sensors, computational power, control system, and mechanics that humans possess. As a result, the types of controllers that need to be created for exoskeletons are quite different from the types of controllers that need to be created for autonomous independent robots.

Exoskeleton development also has a disadvantage over general robot development in that exoskeletons have to work in cooperation with the physiology and biomechanics of the human body. This is a major disadvantage because there is a great deal not understood about the physiology and biomechanics of human movement [4]. If it isn't clear how the metabolic cost of walking is determined by the biomechanical pattern of gait, how is it possible to predict how mechanical assistance will reduce locomotion energetics? If principles governing motor learning during human locomotion are not identified, how can engineers optimize the control algorithms of the exoskeletons? While this disadvantage clearly presents a roadblock to creating useful robotic exoskeletons, there is hope in that studying humans walking with robotic exoskeletons can provide important new insight into human physiology and biomechanics that wasn't previously accessible [4-14].

## Thematic series

In this special thematic series, eight papers contribute new advances on robotic exoskeleton technology and our understanding of how humans respond to mechanical assistance from robotic exoskeletons. Herr starts off with a review on exoskeletons and orthoses, highlighting major accomplishments and discussing future directions in the field [15]. Herr is more conservative than I have been in my prediction of widespread exoskeleton use, as he states it is hopeful that exoskeletons will be in common use by the end of the 21^st ^century. In a second review in the thematic series, Crespo and Reinkensmeyer focus on control strategies that have been used for robotic movement training after neurological injury [16]. The control strategies used for rehabilitation exoskeletons are likely to have a large impact on their success, so this is an area of research that needs substantial effort in the future. Staying in the broader area of rehabilitation exoskeletons, Mankala et al. present a novel exoskeleton design for gait training [17], and Westlake and Patten communicate results from a pilot study on gait training after stroke [18]. In the area of exoskeletons for studying human movement physiology, Sawicki describes a robotic knee-ankle-foot orthosis under proportional myoelectric control [19], and Noel et al. provide some interesting results on adaptation to mechanical forces from a robotic ankle orthosis [20]. The thematic series ends with two excellent contributions on energy harvesting exoskeletons. The first transmits negative mechanical work at the knee into electrical energy [21], and the second uses pneumatics to store energy during stance for powering dorsiflexor assistance during swing [22].

## Conclusion

To advance exoskeleton technology at the fastest rate possible, it is critical that scientists and engineers document and share their successes and failures with the research community. This special thematic series is intended to highlight that need. A major factor limiting the development of powered prostheses in the past has been the lack of carefully controlled scientific studies and open publication of technological advancements. While it is understandable that for-profit companies do not readily publish their research and development work, researchers at universities and institutes need to focus more on how they can move the field forward in cooperation with for-profit companies. University and institute researchers can identify basic principles governing human movement with robotic technologies (exoskeletons, prostheses, etc.) with carefully designed experimental studies. They can also provide unbiased assessment of new technologies with controlled tests using adequate sample sizes. Lastly, they can reach out to for-profit companies working on research and development to offer their services and expertise in mutually beneficial collaborations. While the collaboration work may still not yield peer-reviewed publications, it does provide opportunities for scientists and engineers to learn from each other in a way that would greatly benefit the field.
